# Novel epithelial cell adhesion molecule antibody conjugated polyethyleneimine-capped gold nanoparticles for enhanced and targeted small interfering RNA delivery to retinoblastoma cells

**Published:** 2013-05-06

**Authors:** Moutushy Mitra, Mallikarjuna Kandalam, Judith Rangasamy, Balaji Shankar, Uma K. Maheswari, Sethuraman Swaminathan, Subramanian Krishnakumar

**Affiliations:** 1 Department of Ocular Pathology, Vision Research Foundation, Sankara Nethralaya, Chennai, India; 2 Faculty of Science and Technology, IFHE, Hyderabad, India;; 3 Shanmugha Arts, Science, Technology & Research Academy University, Tanjore, India

## Abstract

**Background:**

Several nanoconjugates have been designed to deliver nucleic acids such as small interfering RNA (siRNA) and DNA to cells to study silencing and expression efficacies. In the present study, we prepared novel epithelial cell adhesion molecule (EpCAM) monoclonal antibody conjugated polyethyleneimine (PEI) capped gold nanoparticles (AuNPs) loaded with EpCAM-specific siRNA molecules to knock-down the *EpCAM* gene in retinoblastoma (RB) cells. We chose EpCAM as a target moiety to deliver siRNA because this molecule is highly expressed in various epithelial cancers and is an ideal target as it is highly expressed in the apical surface of tumor cells while showing basolateral expression in normal cells.

**Methods:**

The EpCAM antibody was conjugated to AuNP-PEI loaded with siRNA molecules to specifically deliver siRNA to EpCAM-expressing RB cells. Conjugation efficiencies were confirmed with ultraviolet-visible spectrophotometry, Fourier transform infrared spectroscopy, and agarose and SDS–polyacrylamide gel electrophoresis. The size and zeta potential were measured using a Zeta sizer analyzer. Nanoparticle internalization and uptake were studied using fluorescent microscopy and flow cytometry. Gene silencing efficacy was monitored with western blot analysis and real-time quantitative PCR.

**Results:**

Optimal size and neutral zeta potential properties of the AuNP-PEI- EpCAM antibody (EpAb) antibody were achieved for the transfection studies. The AuNP-PEI nanoparticles did not show any cytotoxicity to the cells, which means these nanomaterials are suitable for intracellular delivery of siRNA for therapeutic interventions. With EpCAM antibody conjugation, PEI-capped AuNPs loaded with EpCAM siRNA were significantly internalized in the Y79 cells as observed with fluorescence microscopy and flow cytometry and induced a highly significant reduction in the cell viability of the Y79 cells. Through increased binding of EpCAM antibody–conjugated AuNP-PEI nanoparticles, significant downregulation of EpCAM gene was observed in the Y79 cells when compared to the cells treated with the antibody-unconjugated AuNP-PEI nanoparticles.

**Conclusions:**

Thus, a novel antibody conjugated nanocarrier designed to deliver siRNA holds promise as an effective gene therapy strategy for retinoblastoma in the near future. In addition to serving as an siRNA delivery tool for therapy, gold nanoparticles can also serve as imaging modality in diagnosis.

## Introduction

RNA interference has received much interest in biomedical applications as a potential therapeutic option because of the molecules’ advanced ability to knock-down target genes in a specific manner and to overcome the limitations associated with conventional treatments for many diseases [1-4]. Small interfering RNA (siRNA) induces sequence-specific breakdown of complementary messenger RNA (mRNA), leading to inhibition of a target protein at the post-transcriptional level [5,6]. Nonetheless, the use of siRNA in clinical applications has been questioned due to many barriers, including reduced intracellular uptake and severe enzymatic degradation under in vivo circumstances [7,8]. Development of effective delivery transporters is therefore indispensable for siRNA therapy.

Several non-viral polymers [9-11], cationic lipids [12-14], and peptides [15-17] have been used to form nanosized polyelectrolyte complexes via electrostatic interactions with siRNA. In addition, gold nanoparticles (AuNPs) have also been used for intracellular drug delivery [18-22]. AuNPs have also been used for nucleic acid delivery [23-30]. siRNAs were preferred for conjugation on the surface of AuNPs. Later, siRNAs were conjugated to poly(ethylene glycol)-modified AuNPs via biodegradable disulfide linkages [30]. Recently, Wen-Jing et al. used AuNP-PEI for siRNA delivery to target green fluorescent protein expression in MDA-MB-435s cells [31]. However, in the present study we aimed to develop novel antibody conjugated AuNP- polyethyleneimine (PEI) nanoparticles loaded with siRNA molecules to specifically deliver siRNA to tumor antigen-expressing cells. We chose the epithelial cell adhesion molecule (EpCAM) as a target moiety to deliver siRNA because this molecule is highly expressed in various epithelial cancers [32] and is an ideal target as it is highly expressed in the apical surface of tumor cells while showing basolateral expression in normal cells [33]. EpCAM is a 40,000 molecular weight (MW) type I transmembrane glycoprotein that consists of two epidermal growth factor-like extracellular domains, a cysteine-poor region, a transmembrane domain, and a short cytoplasmic tail. Recently, we demonstrated an EpCAM antibody-based targeted approach for enhanced drug delivery to EpCAM-expressing retinoblastoma (RB) Y79 cells using EpCAM antibody conjugated polymeric nanoparticles loaded with chemotherapy drugs [34]. In the present study, we showed that EpCAM antibody conjugated AuNP-PEI nanoparticles loaded siRNA via electrostatic interactions showed greater uptake and enhanced gene silencing efficacy when compared to AuNP-PEI-siRNA without antibody conjugation. Thus, the novel target-specific siRNA delivery system developed in this study may have potential in gene therapy application in the clinical setting.

## Methods

### Materials

Washed tetrakis-hydroxymethyl-phosphonium chloride (THPC) AuNPs and branched polyethyleneimine (BPEI weight-average molecular weight, MW=25 kDa) AuNPs were purchased from nanoComposix, Inc. (San Diego, CA). Reagent 3-(4,5-dimethylthiazol-2-yl)-2,5-diphenyltetrazolium bromide (MTT) was purchased from Sigma-Aldrich (Bangalore, India). The HiPerFect transfection kit was purchased from Qiagen (Santa Clara, CA). Fluorescently labeled 6-fluorescein amidite (FAM)-siRNA, negative control siRNA (NC siRNA), and EpCAM siRNA were obtained from Qiagen. Anti-EpCAM monoclonal antibody was purchased from Santa Cruz Biotechnology (Santa Cruz, CA). Normal immunoglobulin G (IgG) mouse immunoglobulin was obtained from Santa Cruz. Rosewell Park Memorial Institute (RPMI) 1640 media and fetal bovine serum (FBS) were purchased from Gibco-BRL (Rockville, MD). Dithiobis succinimidyl propionate (DSP) and dimethyl sulfoxide (DMSO) were purchased from Sigma-Aldrich.

### Cell culture

The retinoblastoma Y79 cell line was procured from the Cell Bank, RIKEN BioResource Center (Ibaraki, Japan). Y79 was cultured in RPMI 1640 medium supplemented with 10% heat-inactivated fetal calf serum, 2 mM L-glutamine, 0.1% ciprofloxacin, 4.5% dextrose, and 1 mM sodium pyruvate and grown at 37 °C in a 5% CO_2_ humidified incubator (Thermo Fisher Scientific Pvt Ltd. Mumbai, India).

### Electrostatic binding of small interfering RNA to branched polyethyleneimine-capped gold nanoparticles

The molecular weight of the EpCAM siRNA obtained was 14 μg/nmol. BPEI-capped AuNPs were mixed with siRNA at various weight ratios of AuNPs to siRNA (AuNP/siRNA ratios: 0.2, 0.5, 1, 2, and 3) and incubated for 15 min. After incubation, the electrophoretic mobility (15 min at 120 V in TAE buffer; 40 mM Tris/HCl, 1% (v/v) acetic acid, 1 mM EDTA) of the mixture was visualized on 1% (w/v) agarose gel stained with ethidium bromide.

### Conjugation of epithelial cell adhesion molecule antibody to branched polyethyleneimine-gold nanoparticles

Ten mg of PEI-AuNP (branched, MW: 25 kDa) was mixed in 1 ml of DMSO to attain a final concentration of 10 mg PEI/ml. The PEI-AuNP solution was added drop wise to 1 mg of DSP in 1 ml of DMSO. The mixture was incubated for 2 h at room temperature. Later, AuNP-PEI-DSP was coupled to the EpCAM antibody by adding varying nanomole concentrations of the EpCAM antibody (pH 7.4) into the AuNP-PEI-DSP solution containing 100 nmol of PEI [EpCAM-PEI: 0.125 nmol, 0.25 nmol, 0.5 nmol, 0.75 nmol, and 1 nmol of EpCAM antibody against 100 nmol of PEI]. The 100 nmol of PEI corresponded to 2.5 mg of PEI (PEI MW: 25 kDa) and 1 nmol of EpCAM antibody corresponded to 150 μg of antibody (antibody MW: 150 kDa). The reaction suspension was incubated for 2 h at room temperature. Later, the reaction by-products and DMSO were removed by dialyzing against deionized water. Human IgG immunoglobulin conjugated with PEI-AuNP was similarly synthesized and used as negative control for the cell culture studies.

### Small interfering RNA binding of gold nanoparticle-polyethyleneimine- EpCAM antibody

The molecular weight of EpCAM siRNA obtained was 14 μg/nmol. The weight ratio of AuNP:PEI in the solution obtained was 1.6; therefore, 1 ml of the AuNP-PEI preparation contained 0.4 mg of AuNP and 0.25 mg of PEI. AuNP-PEI-EpAb were mixed with siRNA at an AuNPs to siRNA weight ratio of 3 and incubated for 15 min. After incubation, the electrophoretic mobility (15 min at 120 V in TAE buffer; 40 mM Tris/HCl, 1% (v/v) acetic acid, 1 mM EDTA) of the mixture was visualized on 1% (w/v) agarose gel stained with ethidium bromide.

### Characterization of gold nanoparticle-polyethyleneimine conjugates

Transmission electron microscopy observation was performed on a transmission electron microscope with an acceleration voltage of 100 kV. Size distribution and zeta potential analysis of AuNP-PEI and their siRNA and antibody conjugates were performed with dynamic light scattering (DLS) and on a Malvern Zetasizer Nano ZS90 (Malvern Instruments Ltd, Malver, UK). Zeta potential is a measure of the magnitude of the electrostatic or charge repulsion or attraction between particles, and is one of the fundamental parameters known to affect stability. Absorption spectra analyses of the solutions were measured on an ultraviolet-visible spectrophotometry-near infrared (UV-VIS-NIR) spectrometer (Cary 5E, Turun Yliopisto, Finland). Fourier transform infrared spectroscopy (FT-IR) analysis was performed using an IR spectrophotometer 450–4000 cm-1.

### Silencing of the epithelial cell adhesion molecule gene

The Y79 cells (1×10^5^) were seeded in 24-well tissue culture plates and incubated for 24 h to reach about 70% confluence. The cells were then transfected with RPMI medium (0.5 ml) with 10% fetal bovine serum (FBS) containing complexes of HiPerFect reagent and AuNP conjugates (AuNPs or AuNP-PEI or AuNP-PEI-EpAb or AuNP-PEI-siRNA or AuNP-PEI-EpAb-siRNA or naked siRNA of 100 nM) according to the manufacturer’s protocol (Qiagen). After 48 h of incubation, the transfection efficiencies were analyzed. EpCAM silencing efficiency was analyzed with real-time quantitative PCR and western blotting.

### Cellular uptake study

Y79 cells (1×10^5^) were seeded in a 24-well plate and transfected with PEI-AuNPs loaded with FAM-siRNA (50 nmol) or AuNP-PEI-EpAb loaded with FAM-siRNA or free FAM- siRNA (50 nmol) to assess the uptake of AuNPs in the Y79 cells. FAM-siRNA was formulated in PEI-AuNPs as described in the above method and incubated with Y79 cells in 1 ml of RPMI medium for 6 h. Following 6 h of transfection, the cells were harvested, washed, and fixed with 4% formaldehyde, and the slides were mounted and observed under 40× objective using an Axio Observer fluorescent microscope (Carl Zeiss, Berlin, Germany). The EpCAM antibody conjugated AuNP-PEI particles were also detected using fluorescein isothiocyanate (FITC)-conjugated secondary antimouse antibody. Briefly, the Y79 cells were incubated with the EpCAM antibody conjugated AuNP-PEI for 12 h. Then the cells were harvested, washed with PBS (137 mM NaCl, 2.7 mM KCl, 10 mM sodium phosphate dibasic, 2 mM potassium phosphate monobasic and a pH of 7.4), incubated with FITC-labeled antimouse secondary antibody for 2 h, and washed with PBS three times. The cells were fixed on slides, mounted, and observed under a fluorescent microscope. Flow cytometry (FACSCalibur; BD Biosciences, San Jose, CA) analysis was performed to quantify the uptake of the AuNP conjugates in the Y79 cells. The data were analyzed using the CellQuest software program (BD Biosciences).

### 3-(4,5-dimethylthiazol-2-yl)-2,5-diphenyltetrazolium bromide assay for cytotoxicity assessment

To determine the cytotoxicity of the AuNPs/AuNP-PEI, AuNP-PEI-siRNA, and transfection reagents, we performed an MTT assay on the cells transfected with the nanoconjugates. Briefly, 1×10^4^ cells were plated in 96-well flat-bottomed culture plates (100 μl of RPMI medium per well). After 24 h, different materials in complete RPMI medium (100 μl) with serum were added to replace the culture medium, and the Y79 cells were further cultured for 24 h. Following the incubation period, the MTT stock solution in PBS was added to each well except the wells used as blank, to which PBS was added, and the cells were incubated at 37 °C for 4 h. MTT solubilization solution (10% Triton X-100 in acidic isopropanol, 0.1 N HCl) was then added, and the cells were incubated overnight. Colorimetric measurements were made using a spectrophotometer (Beckman Coulter India Private Ltd, New Delhi, India) at 562 nm, and the background was subtracted at 650 nm. The cell viability in the treated wells was normalized to that of the cells treated with PBS.

### Quantitative real-time polymerase chain reaction

RNA was extracted with the guanidine isothiocyanate and chloroform method (TRI Reagent; Sigma-Aldrich) as described in our earlier study [35]. Briefly, cells were harvested from cultures and collected in RNase free vials. To the pellet, 1 ml of TRIzol reagent (Sigma-Aldrich) was added, vortexed for 2 min, and incubated at room temperature for 5 min. Later, 0.5 ml of chloroform was added to the solution, shaken well for 15 s, and centrifuged.

The aqueous layer that contains RNA was transferred to new vials, and 0.5 ml of isopropanol was added and incubated at room temperature for 10 min. After centrifugation, the supernatant was discarded. Then 0.5 ml of 75% ethanol was added, mixed well, and centrifuged. The supernatant was discarded. The pellet was air dried at room temperature for 2 min and reconstituted in 25 μl of RNase-free water. All the centrifugations in RNA extraction were performed at 15,000× *g* for 10 min at 4 °C. All RNA samples were treated with RNase free DNase (Turbo; Ambion, Genetix Biotech Asia Pvt. Ltd., Chennai, India). For all samples, 1 μg of total RNA was used to synthesize first-strand cDNA with reverse transcriptase (SuperScript II; Invitrogen, Joyvel, Chennai, India) and random primers. The cDNA synthesis was performed at 37 °C for 60 min after heat inactivation at 95 °C for 10 min. Gene expression assays for EpCAM (Hs00158980_m1) and two endogenous controls, glyceraldehyde-3-phosphate dehydrogenase (Hs99999905_ml) and HPRT (Hs99999909_m1), were obtained from Applied Biosystems (LabIndia, Chennai, India). Quantification of gene expression was performed in triplicate in a 20 μl volume in 96-well plates on a real-time PCR system (Prism 7300; ABI, Lab India Instruments, Gurgaon, India). Each reaction included 1× primer probe mix (TaqMan; ABI), 1× universal PCR master mix (TaqMan; ABI), and 100 ng of cDNA. The PCR was performed as follows: 2 min at 50 °C, 10 min at 95 °C, and 40 cycles of 15 s at 95 °C, plus 1 min at 60 °C. Commercial software (SDS ver. 1.3; ABI) was used to calculate the ΔΔCt [36] relative expression values for EpCAM normalized to the glyceraldehyde-3-phosphate dehydrogenase endogenous control.

### Western blot analysis

Protein extraction and blotting were performed as described in our earlier study [35]. Briefly, cells were lysed in radioimmunoprecipitation assay (RIPA) lysis buffer for 15 min on ice. An aliquot (100 μg) of lysate was electrophoresed with 10% sodium dodecyl sulfate-polyacrylamide gel and blotted on a nitrocellulose membrane. Membranes were blocked in 5% fat-free milk and then incubated separately with 1:500 diluted mouse monoclonal primary antibody against EpCAM (C-10) overnight at 4 °C. β-actin was used as a loading control (AC-15, dilution: 1:4,000; Sigma). After washing, the membranes were incubated with horseradish peroxidase-conjugated antimouse gamma IgG antibody (diluted to 1:2,000; Santa Cruz Biotechnology) for 1 h at room temperature. The bands were visualized using an enhanced chemiluminescence kit (Amersham, Pittsburgh, PA). Each experiment was performed in triplicate.

### Flow cytometry analysis

The Y79 cells incubated with AuNP-PEI, AuNP-PEI-siRNA (FAM), and AuNP-PEI-EpAb-siRNA (FAM) were harvested, washed, and resuspended in ice-cold PBS (137 mM NaCl, 2.7 mM KCl, 10 mM sodium phosphate dibasic, and 2 mM potassium phosphate monobasic; pH of 7.4), 10% fetal calf serum, and 1% sodium azide. Cells were analyzed on a FACSCalibur flow cytometer (BD Biosciences, San Jose, CA), using the CellQuest software program (BD Biosciences). The fluorescence of the FITC-stained cells was excited with an argon laser at 488 nm, and the emissions were detected at 530 nm.

### Statistical analysis

All experiments were performed in triplicate. The results are reported as mean ± standard deviation. Statistical comparisons were performed using the Student *t* test; p<0.05 was considered statistically significant.

## Results

### Preparation and characterization of polyethyleneimine-capped gold nanoparticles conjugated to small interfering RNA/epithelial cell adhesion molecule antibody

**Figure 1:**
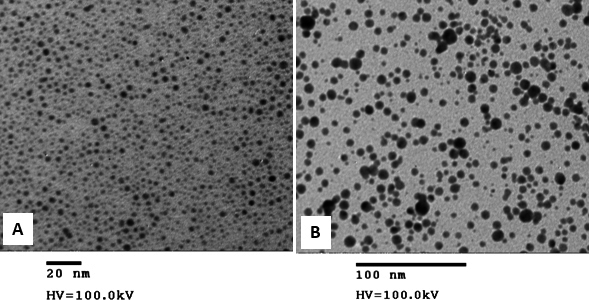
Transmission electron microscopy (TEM) images of gold nanoparticles (AuNP) and polyethyleneimine capped gold nanoparticles (PEI-capped AuNP). The TEM sizing of the AuNPs (**A**) and AuNP-PEI (**B**) were 2.9 nm and 5.8 nm, respectively.

**Figure 2:**
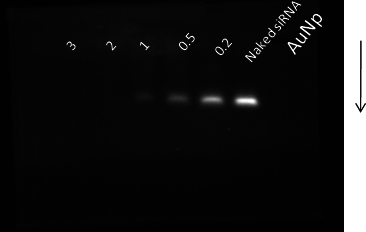
Agarose gel electrophoresis showing the retardation of siRNA loaded AuNP-PEI nanoparticles. The siRNA retardation was observed at a weight ratio of more than 0.5 (AuNP:siRNA).

**Figure 3:**
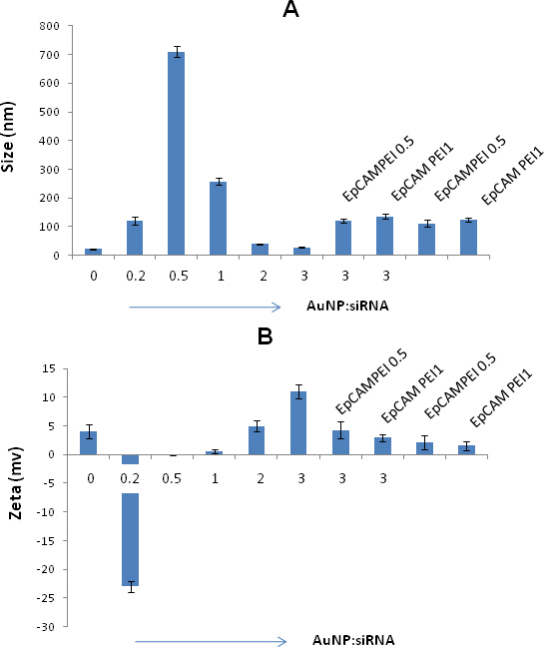
Determination of Size and charge of nanoconjugates. The size distribution of AuNPs mixed with siRNA and/or conjugated EpCAM antibody was determined at varying AuNP:siRNA ratios. **A**: The siRNA conjugated gold nanoparticles showed increased size of about 710 nm and 257 nm at the ratios of 0.5 and 1, respectively. The optimal size of about 28 nm was achieved at the AuNP:siRNA weight ratio of 3:1. The size of the gold nanoparticles conjugated with PEI and EpCAM antibody at a ratio of 0.5 and 1 was 111 and 124, respectively. **B**: The zeta potential measurements of the AuNPs mixed with siRNA and/or conjugated EpCAM antibody. The zeta potential was almost neutral at the weight ratios of 0.5 and 1, which suggests particle aggregation in the solution. The zeta potential was shifted to 5 and 11 at the weight ratios of 2 and 3, respectively. The zeta potential was 2.1 and 1.6 at EpCAMPEI ratio of 0.5 and 1, respectively. The optimal size of about 28 nm was achieved at the AuNP:siRNA weight ratio of 3:1. The size of the AuNP-PEI-EpAb at a ratio of 0.5 and 1 was 111 and 124, respectively. **B**: The zeta potential measurements of the AuNPs mixed with siRNA and/or conjugated EpCAM antibody. The zeta potential was almost neutral at the weight ratios of 0.5 and 1, which suggests particle aggregation in the solution. The zeta potential was shifted to 5 and 11 at the weight ratios of 2 and 3, respectively. The zeta potential was 2.1 and 1.6 at EpCAM-PEI 0.5 and 1, respectively.

We used PEI-capped AuNPs in this study to conjugate siRNA molecules via electrostatic interactions for the cellular delivery. Washed AuNPs were used as the control, and AuNP-PEI was used for the conjugation studies. Typically, the transmission electron microscopy sizing data of the AuNPs and AuNP-PEI demonstrated particle size of 2.9 nm and 5.8 nm, respectively (Figure 1A,B). To demonstrate the siRNA binding ability, AuNP-PEI nanoparticles were mixed with siRNA molecules and analyzed with agarose gel electrophoresis. As shown in Figure 2, siRNA retardation was observed at a weight ratio of more than 0.5 (AuNP:siRNA). This indicates the successful binding of siRNA to AuNP-PEI. As part of the optimization of the weight ratios of AuNP:siRNA, we monitored the size and zeta potential changes on varying weight ratios of AuNP to siRNA. As shown in Figure 3A, we noticed increased size of about 710 nm and 257 nm at the ratios of 0.5 and 1, respectively. The optimal size of about 28 nm was achieved at the AuNP:siRNA weight ratio of 3. The zeta potential was almost neutral at the weight ratios of 0.5 and 1, which suggests particle aggregation in the solution (Figure 3B). However, the zeta potential was shifted to 5 and 11 at the weight ratios of 2 and 3, respectively (Figure 3B).

**Figure 4:**
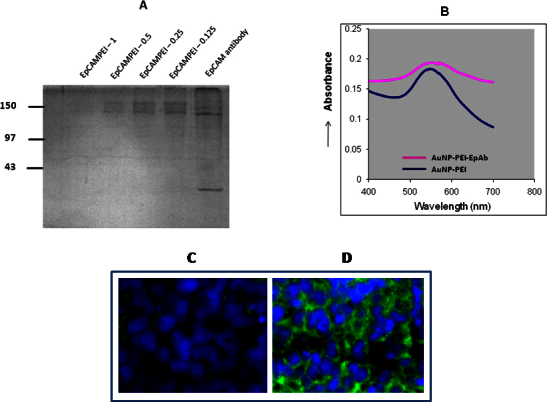
Characterization and validation of EpCAM antibody conjugation to gold nanoparticles. **A**: SDS-PAGE shows retardation of EpCAM antibody conjugated AuNP-PEI nanoparticles. EpCAM antibody retardation was observed along with the increase in EpCAMPEI ratio (0.5 nmol/100 nmol PEI onwards). **B**: A UV-VIS spectrum shows the differences in the peaks between AuNP-PEI and EpCAM conjugated AuNP-PEI nanoparticles. The UV-VIS spectrometry analysis of AuNP-PEI-EpAb (549 nm) demonstrated deviation in the absorption spectra from the typical 529nm spectra of AuNP-PEI.**C**: Fluorescence microscopy image confirms the uptake of EpCAM conjugated AuNP-PEI nanoparticles. The antibody conjugation was detected by FITC labeled anti-mouse secondary antibody in the Y79 cells transfected with AuNP-PEI-EpAb nanoparticles (**D**) compared to cells treated with AuNP-PEI nanoparticles (**C**).

In the second phase, the EpCAM monoclonal antibody was conjugated to the PEI-AuNPs using DSP as a cross-linking agent. At the EpCAM-PEI ratio of 0.5 nmol, we observed retardation of the EpCAM antibody on SDS–polyacrylamide gel electrophoresis analysis as shown in Figure 4A. This indicates that the EpCAM antibody was conjugated to AuNP-PEI nanoparticles. The size of the AuNP-PEI-EpAb at a ratio of 0.5 and 1 was 111 and 124, respectively (Figure 3A). Correspondingly, the zeta potential was 2.1 and 1.6 for EpCAM-PEI 0.5 and 1, respectively (Figure 3B). The EpCAM-PEI ratio (0.5 nmol/100 nmol PEI) was selected for the uptake and gene silencing studies. The UV-VIS spectrometry analysis of AuNP-PEI-EpAb (549 nm) demonstrated deviation in the absorption spectra from the typical 529 nm spectra of AuNP-PEI (Figure 4B). The EpCAM antibody conjugation to AuNP-PEI was also confirmed via the detection of the EpCAM antibody in the Y79 cells using FITC-conjugated antimouse secondary antibody (Figure 4C). Therefore, based on this evidence, it is clear that the EpCAM antibody was conjugated successfully to AuNP-PEI nanoparticles.

### Cytotoxic evaluation of gold nanoparticle conjugates

**Figure 5:**
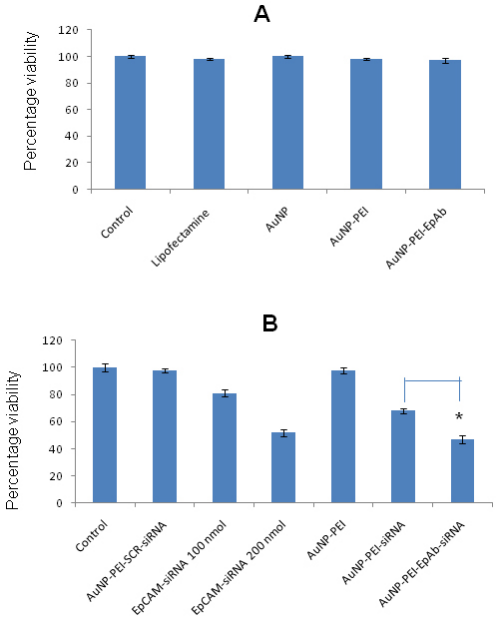
Determination of the effect of nano-conjugates on the cell viability of Y79 retinoblastoma cells. **A**: Cytotoxic evaluation of transfection reagent and gold nanoparticles (AuNP) conjugates. The MTT assay shows no significant cellular cytotoxicity is associated when treated with (gold nanoparticles) AuNP or polyethyleneimine capped gold nanoparticles AuNP-PEI or EpCAM antibody conjugated polyethyleneimine capped gold nanoparticles AuNP-PEI-EpAb (EpCAM:PEI −0.5). **B**: Effect of siRNA loaded AuNP-PEI/AuNP-PEI-EpAb conjugates on Y79 cell viability. The MTT assay showed that the Y79 cells treated with AuNP PEI-EpAb loaded with 100 nmol of siRNA showed significantly decreased viability (47%) when compared with cells treated with AuNP-PEI-siRNA without antibody conjugation (68%; p<0.05). However, there was a significant decrease in the cell viability of the Y79 cells treated with AuNP-PEI loaded with 100 nmol of siRNA when compared with cells treated with100 nmol of naked siRNA (p<0.05).

The MTT assay was performed to assess the cytotoxicity of AuNPs, AuNP-PEI, and AuNP-PEI-EpAb on the Y79 cells. The MTT assay showed that there is no significant cellular cytotoxicity associated when treated with AuNP or AuNP-PEI or AuNP-PEI-EpAb (EpCAM-PEI −0.5; Figure 5A). These data demonstrate that AuNP-PEI nanoparticles are not cytotoxic to cells and could be used for siRNA delivery. To study the effectiveness of siRNA, the Y79 cells were treated with naked EpCAM-siRNA (100 nM), AuNP-PEI-siRNA, and AuNP-PEI-EpAb-siRNA conjugates for 48 h. The MTT assay showed that the Y79 cells treated with AuNP-PEI-EpAb loaded with 100 nmol of siRNA showed significantly decreased viability (47%) when compared with the cells treated with AuNP-PEI-siRNA without antibody conjugation (68%; p<0.05). However, there was a significant decrease in the cell viability of the Y79 cells treated with AuNP-PEI loaded with 100 nmol of siRNA when compared with cells treated with 100 nmol of naked siRNA (Figure 5B; p<0.05). The percentage of viability (52%) observed in the Y79 cells treated with 200 nmol of naked siRNA is equivalent to that of cells treated with AuNP-PEI loaded with 100 nmol of siRNA (N/P ratio of PEI:siRNA is 18:1; Figure 5B).

### Y79 cell uptake of gold nanoparticle conjugates

**Figure 6:**
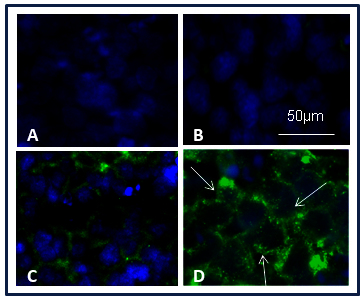
Fluorescence microscope images showing the uptake of siRNA loaded AuNP-PEI/ AuNP-PEI-EpAb nanoparticles. (**A**) control cells; (**B**) Y79 cells treated with naked siRNA; (**C**) Y79 cells treated with AuNP-PEI-siRNA and (**D**) Y79 cells treated with AuNP-PEI- EpAb-siRNA. Free FAM-siRNA was not taken up by the Y79 cells, and therefore, we did not see any detectable fluorescence. In contrast, the Y79 cells demonstrated detectable fluorescence when treated with FAM-siRNA loaded AuNP-PEI (**C**) and AuNP-PEI-EpAb nanoparticles (**D**). Increased fluorescence (white arrows show fluorescence in the Y79 cells) was seen in the Y79 cells due to the EpCAM antibody conjugation to AuNP-PEI-siRNA (FAM) when compared to cells treated with AuNP-PEI-siRNA (FAM) without EpCAM antibody conjugation.

**Figure 7:**
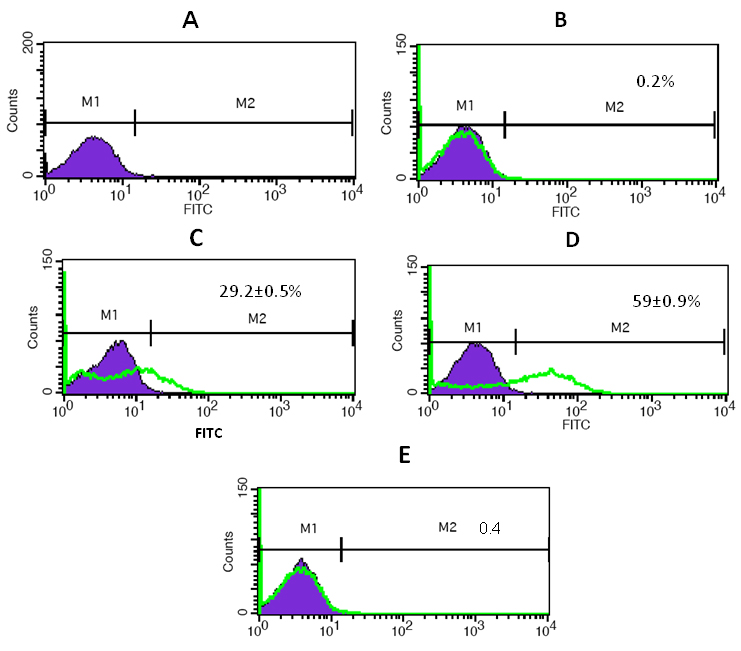
Flow cytometry analysis showing the uptake of siRNA loaded AuNP-PEI/AuNP-PEI EpAb nanoparticles by Y79 cells. (**A**) Control untreated Y79 cells run as control (violet area-M1); (**B**) Y79 cells treated with naked siRNA shows only 0.2% naked siRNA uptake (green peak-M2) when compared to normal untreated Y79 cells (violet area); (**C**) Y79 cells treated with AuNP-PEI-siRNA shows 29.2% siRNA uptake (green peak-M2) when compared to normal untreated Y79 cells (violet area); (**D**) Y79 cells treated with AuNP-PEI-EpAb-siRNA shows 59% of siRNA uptake (green peak-M2) when compared to normal untreated Y79 cells (violet area); (**E**) Only 0.4% uptake of AuNP-PEI-EpAb nanoparticles was observed in EpCAM-siRNA treated Y79 cells. Flow cytometry analysis demonstrated higher uptake of EpCAM conjugated AuNP-PEI-siRNA nanoparticles (59%) by Y79 cells when compared to EpCAM unconjugated AuNP-PEIsiRNA (29.2%; p<0.05).

We used fluorescence microscopy and flow cytometry analysis to investigate the cellular uptake of free FAM-siRNA, AuNP-PEI-siRNA, and AuNP-PEI-EpAb-siRNA. We observed that free FAM-siRNA was not taken up by the Y79 cells, and therefore we did not see any detectable fluorescence (Figure 6B). In contrast, the Y79 cells demonstrated detectable fluorescence when treated with FAM-siRNA loaded AuNP-PEI (Figure 6C) and AuNP-PEI-EpAb nanoparticles (Figure 6D). As expected, we observed increased fluorescence in the Y79 cells due to the EpCAM antibody conjugation to AuNP-PEI-siRNA (FAM) when compared to cells treated with AuNP-PEI-siRNA (FAM) without EpCAM antibody conjugation. Similarly, flow cytometry analysis demonstrated higher uptake of EpCAM conjugated AuNP-PEI-siRNA nanoparticles (59%) by the Y79 cells when compared to EpCAM unconjugated AuNP-PEI-siRNA (29.2%; Figure 7; p<0.05).

### Evaluation of epithelial cell adhesion molecule gene silencing in Y79 cells

**Figure 8:**
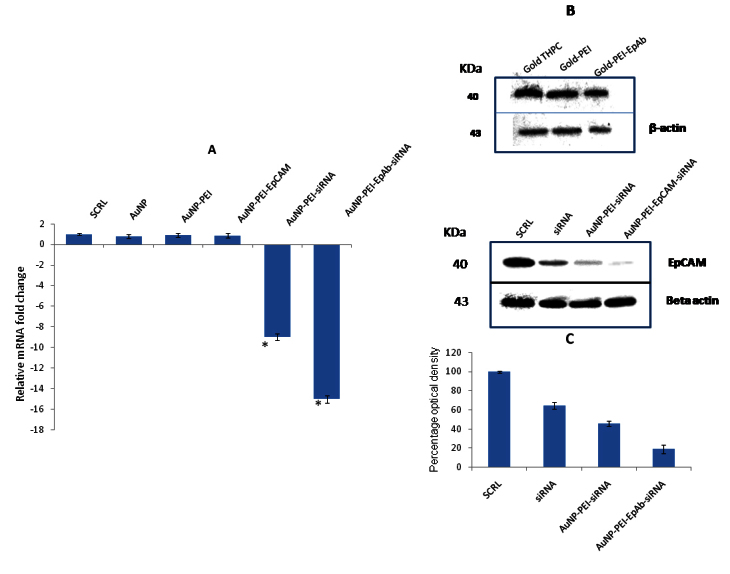
Effect of EpCAM antibody and siRNA conjugated nanoparticles on EpCAM expression in Y79 cells. **A**: Real-time reverse-transcriptase PCR analysis of EpCAM in Y79 cells. The graph shows significant down-regulation of EpCAM mRNA in Y79 cells treated with EpCAM siRNA loaded with polyethylenemine capped gold nanoparticles (AuNP-PEI-siRNA) and EpCAM antibody conjugated siRNA loaded PEI capped AuNP (AuNP-PEI-EpAb-siRNA) when compared to Y79 cells treated with scrambled siRNA or AuNP alone or AuNP-PEI. The asterisk mark represents statistically significant (p<0.05) expression increase in EpCAM compared to other groups. Enhanced downregulation of the EpCAM gene (−15 fold) was observed in the Y79 cells treated with EpCAM-conjugated AuNP-PEI-siRNA when compared with EpCAM-unconjugated AuNP- PEI-siRNA (p<0.05). **B**: Western blotting analysis shows the effect of siRNA loaded AuNP conjugates on the EpCAM protein levels in the Y79 cells. Enhanced knock-down of the EpCAM protein was observed in the Y79 cells when they were treated with EpCAM-conjugated AuNP-PEI-siRNA. β-actin was included as the loading control (panel 2 and 4). **C**: Bar graphs showing the densitometry analysis of western blotting of the EpCAM protein. The graph shows the percentage optical density of the EpCAM protein detected bands on the blotting membrane. Enhanced knockdown of the EpCAM protein was observed in the Y79 cells when they were treated with EpCAM-conjugated AuNP-PEI-siRNA.

Real-time qPCR showed significant downregulation of the *EpCAM* gene (−15-fold) in the Y79 cells treated with EpCAM conjugated AuNP-PEI-siRNA when compared with EpCAM unconjugated AuNP-PEI-siRNA (−9-fold; Figure 8A; p<0.05). The western blot analysis consistently showed enhanced knock-down of the EpCAM protein when treated with AuNP-PEI-siRNA conjugated with the EpCAM antibody (Figure 8B). EpCAM protein expression was unaltered when the Y79 cells were treated with AuNP, AuNP-PEI, and AuNP-PEI-EpAb nanoparticles (Figure 8B).

## Discussion

Positively charged PEI can condense negatively charged nucleic acid siRNA molecules. Therefore, we used PEI-capped AuNPs in this study to conjugate siRNA molecules via electrostatic interactions for the cellular delivery. However, the novelty in this study is to deliver siRNA molecules specifically to tumor cells. This approach requires knowledge of tumor specific proteins, and the protein we used in this study is EpCAM, a transmembrane glycoprotein that is highly expressed in RB tumor cells compared to normal retinal cells.

Therefore, the present study was undertaken to deliver siRNA specifically to EpCAM-expressing tumor cells by conjugating the EpCAM antibody to PEI-capped AuNPs. Initially, conjugated siRNA to PEI-AuNPs and their physicochemical properties were characterized. The siRNA conjugated PEI-capped AuNPs showed higher size distribution at lower AuNP:siRNA ratios, and this could be due to the aggregation of nanoparticles. However, the size distribution of the particles was decreased gradually as the AuNp:siRNA ratios increased (Figure 3A). We optimized the appropriate AuNP-siRNA ratio with the desirable size and charge of the particles for siRNA delivery. Earlier, Wen Jing et al. [31] showed the delivery of siRNA molecules using AuNP-PEI. In the present study, we proposed to deliver siRNA molecules to target-specific cells. In this attempt, the monoclonal EpCAM antibody was conjugated covalently to AuNP-PEI nanoparticles.

To prepare EpCAM-conjugated AuNP-PEI nanoparticles (AuNP-PEI-EpAb), varying EpCAM:PEI molar ratios were synthesized using DSP as a cross-linker that interacts with primary amines on PEI and EpCAM molecules. DSP is a water-insoluble, thiol-cleavable, primary amine-reactive reversible cross-linker [37,38]. As DSP contains a disulfide bond, the bioconjugate synthesized via DSP cross-linking could potentially be cleaved in the highly reducing intracellular environment [39]. This would facilitate the intracellular release of siRNA molecules for gene silencing activity. At the EpCAM:PEI ratio 0.5/100 nmol of PEI, the optimum particles size (111 nm) was observed, and this particular ratio was selected for further siRNA conjugation and downstream experiments. The conjugation efficiency of EpCAM to PEI-AuNPs was characterized with SDS–polyacrylamide gel electrophoresis analysis, FT-IR analysis, and UV-VIS spectrometry techniques. FT-IR and UV-VIS analysis clearly showed characteristic spectrum changes upon EpCAM antibody conjugation to PEI-AuNPs.

Later, we tested the cytotoxic effects of the HiPerFect transfection reagent, AuNPs, AuNP-PEI, and AuNP-PEI-EpAb by incubating the Y79 cells for 24 h. This is essential as the siRNA delivery agents should be minimally cytotoxic to achieve better results in vitro, and it helped us to choose the best combination of nanoparticles for siRNA or gene delivery to the cells in vivo*.* We observed that no significant cellular cytotoxicity was associated when the cells were treated with AuNPs or AuNP-PEI or AuNP-PEI-EpAb. Thus, AuNP-PEI nanoparticles are not cytotoxic to the cells and could be used for siRNA delivery. Before studying the gene knockdown efficiency, we determined the cytotoxic effects of naked siRNA and compared with siRNA loaded EpCAM-AuNP-PEI nanoconjugates. Interestingly, we observed that AuNP-PEI loaded with small amounts of siRNA is as effective as double the amounts of naked siRNA. This would be beneficial for in vivo studies in a way that reduced siRNA amounts will have negligible cytotoxic effects on normal cells in case of non-specific uptake.

Fluorescence microscopy and flow cytometry analysis revealed uptake of FAM-siRNA loaded AuNP-PEI. However, there was a significant increase in fluorescence when the cells were treated with FAM-siRNA loaded AuNP-PEI-EpAb. This confirms two things: One is the EpCAM conjugation to AuNP-PEI nanoparticles, and the second is the EpCAM protein-mediated specific uptake of siRNA loaded AuNP-PEI-EpAb nanoparticles. We observed that the majority of the AuNP-PEI-siRNA conjugates were localized in the cytoplasm of the Y79 cells. This phenomenon would facilitate the release of siRNA effectively in the cytoplasm and therefore effective gene silencing. In addition, EpCAM antibody conjugated AuNP-PEI-siRNA molecules would specifically target EpCAM-expressing cells RB tumor cells and spare the normal cells with low EpCAM levels.

We further evaluated the efficacy of prepared AuNP-siRNA conjugates on EpCAM gene silencing in the Y79 cells with western blotting and real-time quantitative PCR. EpCAM gene silencing with naked siRNA was performed in our earlier study, and we showed that silencing EpCAM leads to a decrease in cell viability and upregulates several genes that induce apoptosis and cell cycle arrest [35]. Earlier, we showed that EpCAM is highly expressed in RB tumor cells when compared to the normal retina [40]. However, non-targeted siRNA therapy in vivo may lead to unwanted damage to the normal retinal cells. Therefore, the present study was undertaken to improve the delivery of siRNA via non-toxic vehicles as well as to deliver siRNA specifically to EpCAM-expressing tumor cells. Earlier, Wen Jing et al. showed effective gene silencing through siRNA delivery using AuNP-PEI nanoparticles [31]. The present study used the concept of targeted delivery by implementing the EpCAM monoclonal antibody conjugation to AuNP-PEI nanoparticles loaded with EpCAM specific siRNA for effective gene silencing. Therefore, AuNP-PEI-EpAb nanoconjugates are nontoxic to cells and could be potential target-specific delivery agents. This concept could very well be used to deliver siRNAs by using tumor-specific antigens expressed on tumor cells. This approach potentially spares the normal cells and induces gene silencing specifically in tumor cells.
